# Genetic risk score has added value over initial clinical grading stage in predicting disease progression in age-related macular degeneration

**DOI:** 10.1038/s41598-019-43144-3

**Published:** 2019-04-29

**Authors:** Thomas J. Heesterbeek, Eiko K. de Jong, Ilhan E. Acar, Joannes M. M. Groenewoud, Bart Liefers, Clara I. Sánchez, Tunde Peto, Carel B. Hoyng, Daniel Pauleikhoff, Hans W. Hense, Anneke I. den Hollander

**Affiliations:** 1 Department of Ophthalmology, Donders Institute for Brain, Cognition and Behaviour, Radboud university medical center, Nijmegen, The Netherlands; 2 Department of Human Genetics, Donders Institute for Brain, Cognition and Behaviour, Radboud university medical center, Nijmegen, The Netherlands; 3 Department of Health Evidence, Radboud University Medical Centre, Nijmegen, The Netherlands; 4 Diagnostic Image Analysis Group, Radboud University Medical Center, Nijmegen, The Netherlands; 5 Department of Ophthalmology, Queens University Belfast, Belfast, United Kingdom; 6 Centre for Public Health, Queen’s University of Belfast, Belfast, United Kingdom; 7 Department of Ophthalmology, St. Franziskus Hospital, Münster, Germany; 8 Institute of Epidemiology and Social Medicine, Westfälische Wilhelms University, Münster, Germany

**Keywords:** Epidemiology, Genetic testing, Predictive markers, Macular degeneration, Medical imaging

## Abstract

Several prediction models for progression of age-related macular degeneration (AMD) have been developed, but the added value of using genetic information in those models in addition to clinical characteristics is ambiguous. In this prospective cohort study, we explored the added value of genetics using a genetic risk score (GRS) based on 52 AMD-associated variants, in addition to the clinical severity grading at baseline as quantified by validated drusen detection software, to predict disease progression in 177 AMD patients after 6.5 years follow-up. The GRS was strongly associated with the drusen coverage at baseline (P < 0.001) and both the GRS and drusen coverage were associated with disease progression. When the GRS was added as predictor in addition to the drusen coverage, R^2^ increased from 0.46 to 0.56. This improvement by the GRS was predominantly seen in patients with a drusen coverage <15%. In patients with a larger drusen coverage, the GRS had less added value to predict progression. Thus, genetic information has added value over clinical characteristics in predicting disease progression in AMD, but only in patients with a less severe disease stage. Patients with a high GRS should be made aware of their risk and could be selected for clinical trials for arresting progression.

## Introduction

Age-related macular degeneration (AMD) is the leading cause of visual impairment of the elderly in the Western World^1,2^. Due to demographic ageing, the prevalence of AMD is expected to grow and by 2040 the global prevalence of AMD is estimated to be 288 million^3^. Therefore, the demand on clinical eye care related to AMD is expected to increase drastically during the next decades.

AMD is a progressive retinal disease in which the early stages are characterized by drusen and pigmentary changes, causing mild visual impairment. In many patients the disease progresses to late stage AMD, which can be distinguished into two subtypes: choroidal neovascularization (CNV) and geographic atrophy (GA), both causing major vision loss. Progression of AMD is often imminent, but the rate of progression differs between cases^4–6^. Unfortunately, factors that determine the rate of progression remain elusive.

AMD is a multifactorial disease, with both environmental and genetic components. The most consistently reported demographic risk factors for AMD are advanced age^7–9^, smoking^7–10^ and high BMI^7,8,11^. Recently, 52 independent genetic variants at 34 genomic loci have been associated with AMD, which together explain approximately half of the heritability^12^. These AMD-associated variants are primarily involved in the modulation of the complement system, lipid metabolism and extracellular matrix remodeling. Thus far, several studies on progression in AMD evaluated only a subset of AMD-associated variants, while the impact of all 52 genetic variants on disease progression has not yet been studied^13–19^.

Several prediction models for disease progression in AMD have been developed, but the added value of using genetic information in these models is ambiguous^20^. Indeed, clinical disease stage at baseline was shown to be one of the strongest predictors of progression^4,5,8,21^. Moreover, a recent prospective cohort study concluded that, when the AMD severity at baseline is included as predictor for progression to late stage AMD, the genetic risk score (GRS) offers limited additional predictive power to the model^15^.

While it is intuitive that a more severe phenotype at baseline would progress more rapidly than a milder phenotype, we hypothesized that a GRS could still be informative for patients and ophthalmologists, in particular in earlier disease stages. This study therefore investigated the association between the GRS and disease progression in patients with non-advanced AMD. In addition, we explored in which patients a GRS has added value for predicting progression besides clinical severity grading at baseline.

## Results

A total of 397 participants with non-advanced AMD in the worst eye at baseline were selected from the Muenster Aging and Retina Study (MARS), a prospective observational cohort study from Muenster, Germany. Of these, 213 participants underwent follow-up examination (mean follow-up time 6.5 years, 95% Confidence Interval (CI) 6.4–6.8 years) and 184 patients were lost to follow-up because of the following reasons: declining further participation (n = 101), death (n = 16), developing an eye condition precluding grading (n = 7) or unknown reason (n = 60). Participants who did not visit follow-up were significantly older (mean difference of 1.4 years) and more often had a history of smoking (mean difference of 13.8%) (Supplementary Table S1). Of the 213 participants with a follow-up examination, DNA was available for 177 participants for genotyping and GRS calculation, and were included into the statistical analyses.

**Figure 1:**
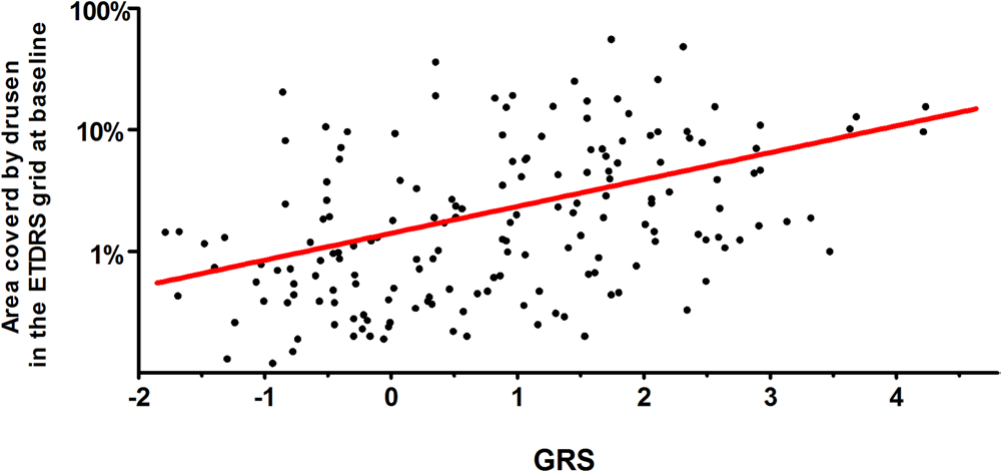
Association between GRS, as based on 52 AMD variants, and the drusen coverage at baseline in the 177 participants of the MARS study with non-advanced AMD at baseline. The red line represents the trend line of the GRS with the area covered by drusen in the ETDRS grid at baseline (β = 1.57, 95% CI 0.75–2.39, P < 0.001). AMD Age related Macular Degeneration; ETDRS Early Treatment Diabetic Retinopathy Study; GRS Genetic Risk Score; MARS Muenster Aging and Retina Study.

First, participants were stratified into three groups as based on the drusen area in the Early Treatment Diabetic Retinopathy Study (ETDRS) grid at baseline. The categories were: <1%, coverage, 1–10% coverage and greater than 10% coverage as specified previously^22,23^. Table 1 shows the characteristics of participants in the three drusen coverage categories. Since a positive trend in mean GRS over the three drusen coverage categories was observed, we analyzed the association between the GRS and drusen coverage at baseline as a continuous measure. As seen in Fig. 1, the GRS was significantly associated with the drusen coverage within the ETDRS grid (β = 1.57, 95% CI 0.75–2.39, P < 0.001).

**Table 1:** Characteristics of patients between drusen area categories, as based on the drusen coverage in the ETDRS grid at baseline.

	<1% drusen coverage (n = 73)	1–10% drusen coverage (n = 79)	>10% drusen coverage (n = 25)	P value
Age, mean (SD), years	70.1 (5.3)	71.0 (5.0)	70.5 (4.7)	0.54
Sex				0.022
Female (n (%))	34 (46.6%)	53 (67.1%)	17 (68.0%)	
Male (n (%))	39 (53.4%)	26 (32.9%)	8 (32.0%)	
BMI, mean (SD), kg/m^2^	27.2 (4.3)	26.5 (3.9)	26.3 (3.6)	0.49
Smoking history				0.16
Current smoking (n (%))	4 (6.3%)	7 (9.7%)	1 (4.5%)	
Smoked in the past (n (%))	22 (34.4%)	21 (29.2%)	2 (9.1%)	
Never smoked (n (%))	37 (58.7%)	44 (61.1%)	19 (86.4%)	
Non-advanced AMD stage				<0.001
Early AMD (n (%))	55 (75.3%)	43 (54.4%)	5 (20.0%)	
Intermediate AMD (n (%))	18 (24.7%)	36 (45.6%)	20 (80.0%)	
GRS, mean (SD)	0.98 (1.00)	1.10 (1.32)	1.72 (1.37)	<0.001

The non-advanced AMD stage was graded with the AREDS basic clinical classification scale. The GRS includes 52 AMD-associated variants.

AMD Age related Macular Degeneration; AREDS Age-Related Eye Disease Study; AMD Age related Macular Degeneration; BMI Body Mass Index; ETDRS Early Treatment Diabetic Retinopathy Study; GRS genetic risk score; SD Standard Deviation.

### The drusen coverage and disease progression

**Figure 2:**
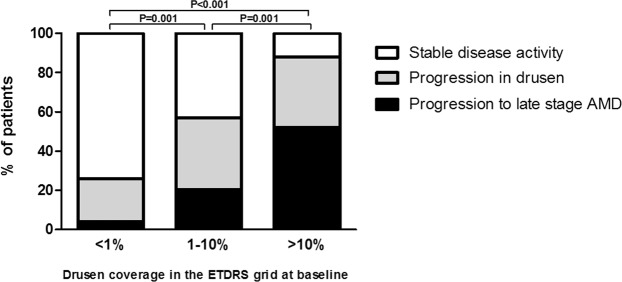
Percentage of participants from the MARS study with stable disease activity, progression in drusen and progression to late stage AMD after 6.5 years, as stratified by the percentage of drusen coverage in the ETDRS grid at baseline. AMD Age related Macular Degeneration; ETDRS Early Treatment Diabetic Retinopathy Study.

Next, we compared progression rates after 6.5 years between the three drusen coverage groups and determined whether the disease remained stable, showed an increase in drusen or progressed to late stage AMD (Fig. 2). As expected, the majority of participants with a drusen coverage <1% at baseline remained stable in their disease activity (74%), while patients with a drusen coverage between 1–10% and >10% at baseline were less likely to remain stable (43% and 12%, respectively). We observed that an increase in drusen over 6.5 years was most likely in participants with a drusen coverage between 1–10% (37%) compared to patients with a drusen coverage <1% and >10% (22% and 36%, respectively). Progression to late stage AMD was most often observed in patients with a drusen coverage >10% compared to a drusen coverage of <1% and 1–10% (52% vs 4% and 20%, respectively).

### The GRS and disease progression

**Figure 3:**
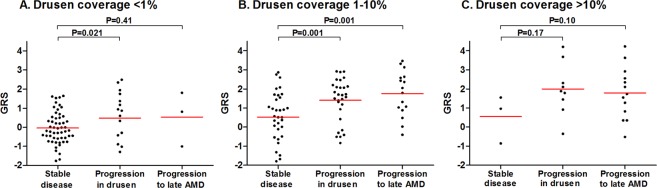
Genetic risk score (GRS) of participants who were stable in their disease (no progression), had progression in drusen, or progression to late stage AMD after 6.5 years follow-up in participants with (**A**) drusen coverage <1% at baseline; (**B**) drusen coverage between 1–10% at baseline; and (**C**) drusen coverage >10% at baseline; in the ETDRS grid. Red lines represent mean GRS per subgroup. AMD Age related Macular Degeneration; ETDRS Early Treatment Diabetic Retinopathy Study; GRS Genetic Risk Score.

Then we analyzed the influence of the GRS on disease progression in each drusen area category. As seen in Fig. 3A, in the group with a drusen coverage <1%, participants with progression in drusen had a higher mean GRS when compared to participants who were stable in their disease (P = 0.021) and no significant difference was observed between the mean GRS and progression to late stage AMD in that category (P = 0.41). In the 1–10% drusen coverage group, shown in Fig. 3B, mean GRS was higher in patients with progression in drusen (P = 0.001) as well as in patients who progressed to late stage AMD (P = 0.001) when compared to patients who were stable in their disease. In the group with a drusen coverage >10%, as presented in Fig. 3C, mean GRS did not differ between patients with stable disease and patients with progression in drusen (P = 0.17) or progression to late stage AMD (P = 0.10).

### The added value of the GRS in predicting disease progression

To further evaluate the added value of the GRS in addition to drusen coverage at baseline to predict disease progression in AMD, we compared the R^2^ of different prediction models in the entire study (Table 2). When only the socio-demographic variables and clinical grading stage (early or intermediate AMD) were used to predict AMD progression (model A), the model had a moderate predictive value (Nagelkerke R^2^ 0.39; AIC 270.3). Adding drusen coverage at baseline alone to the prediction model showed noticeable improvement (model B, Nagelkerke R^2^ 0.46; AIC 262.7). Next, adding a minimal GRS consisting of five major genetic AMD variants in *CFH* (rs10922109 and rs570618), *CFB* (rs116503776)*, ARMS2/HTRA1* (rs3750846) and C3 (rs2230199), (model C) produced also a significantly better predicting performance compared to model A (Nagelkerke R^2^ 0.48; AIC 258.0). Then, using all 52 AMD variants in the maximal GRS (model D), produced obviously better prediction performance compared to model C (Nagelkerke R^2^ 0.52; AIC 249.6). Finally, when the drusen coverage at baseline and the maximal GRS were both added to predict progression, the predicting performance further increased in comparison to all other four models (model E, Nagelkerke R^2^ 0.56; AIC 241.9).

**Table 2:** Prediction models for disease progression in AMD using demographic characteristics and clinical grading stage, drusen coverage at baseline and genetic risk score (GRS) as model predictors, as analyzed with multinomial logistic regression.

Model	Model predictors	Nagelkerke R^2^	Cox and Snell R^2^	McFadden R^2^	AIC
Model A	Age, sex, smoking history, BMI, non-advanced AMD stage	0.39	0.34	0.20	270.3
Model B	Age, sex, smoking history, BMI, non-advanced AMD stage, drusen coverage at baseline	0.46	0.40	0.25	262.7
Model C	Age, sex, smoking history, BMI, non-advanced AMD stage, minimal GRS (5 major AMD variants)	0.48	0.42	0.27	258.0
Model D	Age, sex, smoking history, BMI, non-advanced AMD stage, maximal GRS (52 AMD variants)	0.52	0.45	0.29	249.6
Model E	Age, sex, smoking history, BMI, non-advanced AMD stage, drusen coverage at baseline, major GRS (52 AMD variants)	0.56	0.49	0.33	241.9

The non-advanced AMD stage is subdivided into early AMD and intermediate AMD, as graded with the AREDS basic clinical classification scale. The drusen coverage at baseline is the percentage of drusen in the ETDRS grid in the worst eye at baseline, as determined by drusen detection software with the RetCAD. The minimal GRS includes the five major genetic AMD variants in *CFH* (rs10922109 and rs570618), *CFB* (rs116503776) *ARMS2/HTRA1* (rs3750846) and *C3* (rs2230199), whereas the maximal GRS includes 52 AMD-associated variants.

AIC Akaike information criterion; AREDS Age-Related Eye Disease Study; AMD Age related Macular Degeneration; BMI Body Mass Index; GRS Genetic Risk Score; RetCAD Retina computer-aided detection system.

**Figure 4:**
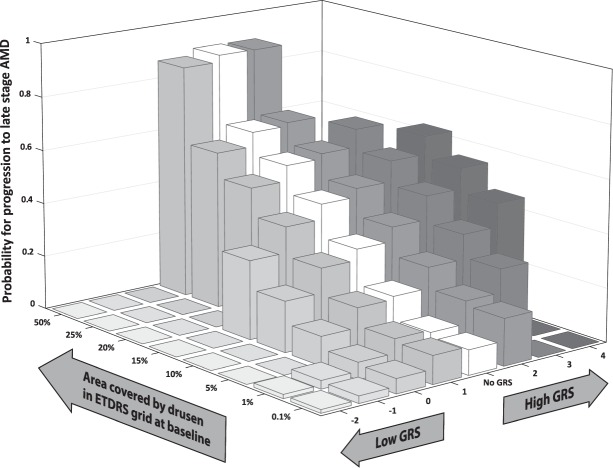
Probability for progression to late stage AMD after 6.5 years, as determined by the GRS including 52 AMD-associated variants, and the drusen coverage in the ETDRS grid at baseline based on multinomial logistic regression. Each bar indicates the probability for progression to late stage AMD after 6.5 years as determined by the combination of drusen coverage and GRS; The white bars indicate the probabilities for progression if GRS was not determined and only the drusen coverage at baseline was used as predictor. Bars were not visualized in case combinations of GRS and drusen coverage at baseline were improbable and not found in our dataset. AMD Age related Macular Degeneration; ETDRS Early Treatment Diabetic Retinopathy Study; GRS Genetic Risk Score.

To illustrate the relationship between both GRS and drusen coverage at baseline for progression, we modeled the probabilities for progression to late stage AMD after 6.5 years, stratifying for GRS and drusen coverage at baseline (Fig. 4). Bars were not visualized in case combinations of GRS and drusen coverage at baseline were improbable and not found in our dataset. The white bars indicated progression probabilities when only the drusen coverage at baseline was used as predictor. The probabilities of progression to late stage AMD were most influenced by the GRS in patients with a drusen area between 1% and 15% of the ETDRS grid. When only the drusen area was determined in these patients, progression probabilities ranged between 10.5% to 44.9%, whereas if the GRS was taken into account, probabilities for progression ranged between 1.4% to 62.3%. In patients with a larger drusen coverage at baseline, the GRS had less added value to change the probability in AMD progression.

## Discussion

This prospective cohort study analyzed the association between the GRS and progression in AMD after 6.5 years of follow-up in patients with non-advanced AMD and explored in which patients a GRS has additive value for predicting disease progression in addition to clinical grading stage.

The GRS was significantly associated with the total drusen coverage in the ETDRS grid. Drusen coverage was associated with disease progression, in particular for progression to late stage AMD. Mean GRS was higher in participants with progression in drusen and to late stage AMD, but only in groups with a drusen coverage of <1% and between 1–10%. To investigate if the GRS had added value in predicting progression in addition to the clinical grading stage at baseline, we compared the explained variances between these predictors. When the GRS was added as predictor in addition to the drusen coverage at baseline, Nagelkerke R^2^ increased from 0.46 to 0.56. The improvement of prediction in disease progression caused by the GRS was mostly seen in patients with a drusen area <15% of the ETDRS grid. In patients with a larger drusen area, the GRS had less effect to change the probability in progression.

Mean GRS was higher in patients with progression in drusen or progression to late stage AMD, but only in groups with a drusen coverage of <1% and between 1–10%. Multiple studies have also shown that AMD-associated genetic variants are associated with disease progression, but in most studies only a subset of AMD-associated variants were used^13–19^. In addition, the total number of AMD-associated variants is also known to be associated with the clinical disease stage, which was also confirmed in our study where the GRS was associated with the drusen coverage^16,24^. According to a recent prospective cohort, genetic variants only provide a limited additional predicting power when the clinical grading stage at baseline is included as predictor^15^. However, in our study, the explained variance increased from 0.46 to 0.56 when the maximal GRS including 52 AMD variants was added as predictor in addition to the clinical grading stage. So, despite the fact that the clinical stage at baseline is a very strong predictor for disease progression, we showed that the GRS can have added value over the clinical grading stage, especially when both major and minor genetic AMD variants are included.

The added value of the GRS over the clinical grading stage at baseline was mainly seen in patients with a drusen area <15%. When only the drusen area was considered as predictor, the probability of progression to late stage AMD in patients with drusen coverages between 1% and 15% could be both over- and underestimated within a range of 1.4% to 62.3% depending on a patient’s GRS. Thus, GRS information in these patients effectively stratified them into fast and slow progressors, even though they had very similar baseline clinical characteristics. In patients with a larger drusen area, the GRS had less added value for predicting progression, partly because participants could not be stratified based on their GRS. Progression in these patients was mainly determined by the clinical grading stage at baseline.

This study and other studies^4,5,8,15,21^ have shown that the clinical grading stage at baseline is associated with progression in AMD, in particular for progression to late stage AMD. Drusen are known to be early signs of AMD and increase in area over time before late stage AMD develops^25^. Because a larger drusen area increases the probability for developing late stage AMD, patients who have rapid drusen accumulation are also more likely to progress faster towards late stage AMD.

Currently, classification of the clinical grading in AMD is mainly performed manually. However, human observer classification is time-consuming and prone to inter-observer variation. Therefore, automatic classification detection and quantification systems are being developed, which have many advantages^26–28^. It may allow cost-efficient screening in the clinic, but can also help to classify patients in research programs for evaluating disease progression, as was used in this prospective cohort. We demonstrated that drusen coverage at baseline, automatically quantified in a continuous measure, can be used to predict disease progression in AMD.

In addition to the usefulness of automatic detection software for disease stage classification in predicting progression, genetic testing has added value, but only in patients with a less severe disease stage at baseline. In the clinic, patients with a high GRS could be made more aware of their risk and motivated to make lifestyle changes to slow down disease progression. Secondly, clinical trials often select patients with a late clinical grading stage. However, it could be argued that interventions at these stages may be too late. Using a GRS would allow for the recognition of fast progressing individuals at an early disease stage. These patients would be ideal participants for clinical trials investigating drugs that arrest disease progression from early to late stages. In the future, ophthalmologists may use a combination of socio-demographic, retinal phenotype and genetic information to recognize and treat those patients who are at risk of rapid progression before they reach the late vision-threatening stages in AMD.

This prospective cohort study has several strengths. First of all, to our knowledge the current study is the first to use a GRS based on all 52 most recently discovered AMD variants^12^. Therefore, this study provides a more comprehensive measurement of the overall genetic contribution to disease progression in AMD compared to other studies^13–19^. Secondly, we used reliable detection software for detailed and accurate drusen quantification on the color fundus photographs which offered us more accuracy in the analysis compared to other studies that used manual grading protocols^5,23,29^. Third, in other cohorts the disease stage was not stratified in the analysis, as a result of which the researchers concluded that genetic testing has only a small additional power^15^. However, in our study we explored the added value of the GRS over the clinical grading stage at baseline, by stratifying the drusen area and showed that genetic testing can be useful to predict progression, but only in patients with a less severe disease stage. Finally, instead of only analyzing progression to late stage AMD, the current study also evaluated progression in drusen, providing more robust results between the GRS and disease progression in AMD^15,30,31^.

However, this study also has some limitations. Our study has a relatively large number of participants who were lost to follow-up. DNA was only genotyped for participants with a follow-up measurement and therefore we do not know the genetic contribution for those who dropped out during the study. In addition, the 52 AMD variants were not directly genotyped, but based on imputation of a global screening array, which might have caused small deviations in the accuracy of the GRS. Finally, although we present a sufficiently sized cohort, it is relatively small compared to other AMD cohorts^4,13,15,32–34^. Therefore, replication of our findings in other studies is warranted to confirm the value of genetic testing in addition to the clinical grading stage at baseline in predicting progression in AMD.

To conclude, the genetic risk score (GRS) has added value over clinical grading stage at baseline in predicting disease progression, in particular in patients with a less severe disease stage. Without a determination of the GRS in these patients, probabilities of disease progression are either under- or overestimated. If confirmed, our findings can be used in the clinic for genetic consultation in disease progression and can be used in clinical trials to identify patients with risk of rapid disease progression of AMD at earlier disease stages.

## Methods

### Study population

Participants were selected from the Muenster Aging and Retina Study (MARS), a prospective observational cohort study from Muenster, Germany. The study was approved by the Institutional Review Board of the University of Muenster and performed according to the standards of the Declaration of Helsinki. AMD patients and controls between 60 to 80 years were recruited by ophthalmologists in Muenster and identified by reviewing patient records from a retina specialty clinic (St. Franziskus Hospital, Muenster, Germany). After providing informed consent, participants were examined at baseline (June 2001 to October 2003, MARS-I), and during a follow-up examination (February 2007 to March 2010, MARS-III). Demographic data, such as gender, age, BMI and smoking history were obtained at baseline and 30 degrees stereoscopic digital color fundus photographs were taken of the macula of both eyes at both visits.

### Grading

The stereoscopic digital color photographs from baseline and follow-up visits were graded according to the Age-Related Eye Disease Study (AREDS) basic clinical classification scale by experienced graders in a certified grading center (Belfast Reading Centre, Belfast, Ireland)^30,31^. No AMD was classified as having no retinal abnormalities or only small drusen <63 µm without pigment abnormalities; early AMD was classified as having drusen >63 µm and <125 µm without pigment abnormalities; intermediate AMD was classified as having drusen >125 µm with or without pigment abnormalities; and late stage AMD was classified as having choroid neovascularization (CNV) or geographic atrophy (GA) > 175 µm. In addition, when no late stage AMD had developed at the follow-up visit, the graders determined if clinical progression was present caused by a change in number of drusen or drusen size (indicated as progression in drusen) or if the severity of the disease was comparable with the baseline visit (indicated as stable disease activity), as based on their clinical expertise.

### Drusen quantification

To analyze the drusen area at baseline, drusen were quantified by analyzing the color fundus images with the Retina computer-aided detection system RetCAD v.1.2.0 (Thirona, Nijmegen, The Netherlands). The RetCAD is a validated detection software for drusen quantification based on deep learning^32^. Anatomical structures including the optic disc and fovea were automatically detected and manually reviewed for proper placement of the Early Treatment Diabetic Retinopathy Study (ETDRS) grid. Quantification was done based on a threshold drusen probability map of 0.9, which is used to generate a binary drusen map to calculate the drusen area. Drusen coverage was thereafter calculated as a percentage of the total ETDRS grid. The study eye was thereafter selected based on the eye with the highest drusen coverage in the ETDRS grid at baseline, as determined by the RetCAD.

### Genetic Risk Score

DNA was extracted from venous blood samples, which were collected at the baseline visit. Patients of whom follow-up data at MARS-III was available were genotyped for 660,000 single nucleotide polymorphisms (SNPs) using the Infinium Global Screening Array (GSA) chip (Illumina, San Diego, USA). Quality control checks were done on the data using PLINK (v1.9)^33^. Samples and variants with more than 5% missing data were excluded from the dataset. Following quality control, genotyping data was imputed using the Michigan Imputation Server with reference panel HRC (v1.1)^34^. After imputation, 44 out of 52 AMD-associated variants described by Fritsche *et al*.^12^, were extracted from the dataset^12^. LDlink was used to find substitution variants that were in high linkage disequilibrium (LD) with 8 AMD variants that were not directly present in the dataset (Supplementary Table S2)^35^.

Finally, we computed a weighted genetic risk score (GRS) of the 52 AMD variants, with weights determined by their estimated effect size. For each participant, $GRS={\sum }_{i=1}^{52}({G}_{i}{\beta }_{i})$, where *β*_*i*_ is the natural logarithm of the odds ratio (OR) of the minor allele of variant *i* as provided in the original discovery genome wide association study (GWAS) of the International AMD Genomics Consortium (IAMDGC) and *G*_*i*_ is the corresponding genotype (coded as 0: no minor allele; 1: one copy of minor allele; 2: two copies of minor allele)^12,36^.

### Statistical analyses

Participants with non-advanced AMD (early or intermediate AMD) in the worst eye at baseline with available genotypes for 52 AMD-associated variants and a follow-up visit, were included in the statistical analyses. All data analyses were performed with SPSS for Windows version 22 (SPSS IBM, New York, USA). Multivariate general linear modeling was used to investigate differences in mean GRS between the three progression groups. Multinomial logistic regression was used to compare progression rates between drusen area categories, to build multiple prediction models, and to model the progression probabilities for late stage AMD. To investigate differences in baseline characteristics between drusen area categories and for performing drop-out analyses, we used ANOVA and/or independent sample t-tests (with P < 0.05 as the significance threshold). All progression analyses were adjusted for age, sex, BMI, smoking history and AMD severity of the worst eye at baseline.

## Supplementary information

Supplementary Tables

## Data Availability

The datasets generated and/or analyzed during the current study are available from the corresponding author on reasonable request.
