# Supplementary material for: Genetic risk score has added value over initial clinical grading stage in predicting disease progression in age-related macular degeneration
Source: Sci Rep. 2019 Apr 29;9:6611. doi: 10.1038/s41598-019-43144-3 (PMC6488669; doi:10.1038/s41598-019-43144-3)
Supplement: Supplementary file 1 — Supplementary Tables [file 41598_2019_43144_MOESM1_ESM.pdf]

Genetic risk score has added value over initial clinical grading stage in predicting disease progression in age-related macular degeneration

Thomas J. Heesterbeek, Eiko K. de Jong, Ilhan E. Acar, Joannes M.M. Groenewoud, Bart Liefers, Clara I. Sánchez, Tunde Peto, Carel B. Hoyng, Daniel Pauleikhoff, Hans W. Hense, Anneke I. den Hollander

Supplementary tables

Supplementary Table S1. Comparison of demographic variables between participants from the MARS cohort with a follow-up visit (n=213) and participants who dropped out (n=184)

	**Follow-up** (n=213)	**Drop-out** (n=184)	**P value**
Age, mean (SD), years	70.4 (5.1)	71.8 (5.5)	0.011
Sex			0.49
Female (n (%))	127 (59.6%)	116 (63.0%)	
Male (n (%))	86 (40.4%)	68 (37.0%)	
BMI, mean (SD), kg/m^2	26.6 (4.0)	27.0 (4.3)	0.31
Smoking history			0.025
Current smoking (n (%))	12 (6.3%)	18 (11.4%)	
Smoked in the past (n (%))	58 (30.5%)	62 (39.2%)	
Never smoked (n (%))	120 (63.2%)	78 (49.4%)	
Non-advanced AMD stage			0.29
Early AMD (n (%))	127 (59.6%)	100 (54.3%)	
Intermediate AMD (n (%))	86 (40.4%)	84 (45.7%)	

The non-advanced AMD stage was graded with the AREDS basic clinical classification scale.
AMD Age related Macular Degeneration; AREDS Age-Related Eye Disease Study; BMI Body Mass Index; MARS Muenster Aging and Retina Study; SD Standard Deviation

Supplementary Table S2. Extracted 52 AMD variants from imputed genotyping data used for calculating the GRS in the 177 participants of the MARS cohort with non-advanced AMD at baseline

Chr	Locus Name	Variant	Substitution variant	LD		Major/Minor allele	MAF	β minor allele
				R^2	D'			
1	*CFH*	rs10922109				C/A	.331	-0.67
	CFH	rs570618				G/T	.446	0.55
	CFH	rs121913059				C/T	.000	3.86
	CFH	rs148553336				T/C	.011	-1.17
	CFH	rs187328863	rs79524406	0.954	0.984	C/T	.025	0.39
	CFH	rs61818925	rs12142766	0.850	0.982	G/T	.282	0.17
	CFH	rs35292876				C/T	.008	0.43
	CFH	rs191281603				C/G	.006	-0.89
2	*COL4A3*	rs11884770				C/T	.305	-0.09
3	*ADAMTS9-AS*	rs62247658				T/C	.435	0.13
	COL8A1	rs140647181				T/C	.017	0.62
	COL8A1	rs55975637				G/A	.153	0.15
4	*CFI*	rs10033900				C/T	.517	0.14
	CFI	rs141853578				C/T	.000	1.63
5	*C9*	rs62358361				G/T	.025	0.51
	PRLR/SPEF2	rs114092250				G/A	.037	-0.34
6	*C2/CFB/SKIV2L*	rs116503776				G/A	.093	-0.67
	C2/CFB/SKIV2L	rs144629244				G/A	.008	1.03
	C2/CFB/SKIV2L	rs114254831				A/G	.285	0.12
	C2/CFB/SKIV2L	rs181705462				G/T	.011	0.45
	VEGFA	rs943080				T/C	.475	-0.14
7	*KMT2E/SRPK2*	rs1142				C/T	.350	0.13
	PILRB/PILRA	rs7803454				C/T	.167	0.14
8	*TNFRSF10A*	rs79037040				T/G	.449	-0.11
9	*MIR6130/RORB*	rs10781182				G/T	.260	0.11
	TRPM3	rs71507014	rs10868899	0.708	0.992	GC/G	.500	0.10
	TGFBR1	rs1626340				G/A	.192	-0.13
	ABCA1	rs2740488				A/C	.254	-0.11
10	*ARHGAP21*	rs12357257				G/A	.212	0.11
	ARMS2/HTRA1	rs3750846				T/C	.308	1.08
12	*RDH5/CD63*	rs3138141				C/A	.246	0.17
	ACAD10	rs61941274				G/A	.002	0.47
13	*B3GALTL*	rs9564692				C/T	.277	-0.10
14	*RAD51B*	rs61985136	rs1028577	0.966	0.992	T/C	.390	-0.13
	RAD51B	rs2842339				A/G	.116	0.17
15	*LIPC*	rs2043085				T/C	.356	0.14
	LIPC	rs2070895				G/A	.254	-0.15
16	*CETP*	rs5817082	rs1864163	0.994	1.0	C/CA	.215	-0.14
	CETP	rs17231506				C/T	.359	0.10
	CTRB2/CTRB1	rs72802342				C/A	.051	-0.24
17	*TMEM97/VTN*	rs11080055				C/A	.562	-0.09
	NPLOC4/TSPAN10	rs6565597				C/T	.373	0.11
19	*C3*	rs2230199				C/G	.249	0.39
	C3	rs147859257				T/G	.003	1.17
	C3	rs12019136				G/A	.048	-0.30
	CNN2	rs67538026	rs8102732	0.688	0.880	C/T	.497	-0.10
	APOE	rs429358				T/C	.150	-0.40
	APOE	rs73036519				G/C	.294	-0.10
20	*MMP9*	rs142450006	rs3859613	0.508	0.984	TTTTC/T	.263	-0.17
	C20orf85	rs201459901	rs76592054	0.958	0.990	T/TA	.079	-0.28
22	*SYN3/TIMP3*	rs5754227				T/C	.133	-0.24
	SLC16A8	rs8135665				C/T	.172	0.13

After imputation of the genotyping data, 44 out of 52 AMD-associated variants were extracted from the dataset. LDlink was used to find substitution variants that were in high LD with 8 AMD variants that were not directly present in the dataset. β's were computed using the natural logarithm of the ORs of the minor alleles of the 52 AMD-associated variants, as provided in the original discovery GWAS of the IAMDGC.

A Adenine; AMD Age related Macular Degeneration; C Cytosine; Chr chromosome; G Guanine; GRS Genetic Risk Score; GWAS genome wide association study; IAMDGC International AMD Genomics Consortium; LD Linkage Disequilibrium; OR Odds Ratio; MAF minor allele frequency; MARS Muenster Aging and Retina Study; T Thymine
